# Immunogenicity of imported foot-and-mouth vaccines in different species in Mongolia

**DOI:** 10.1016/j.vaccine.2019.12.053

**Published:** 2020-02-11

**Authors:** Gerelmaa Ulziibat, Odonchimeg Maygmarsuren, Bodisaikhan Khishgee, Ganzorig Basan, Batkhuyag Sandag, Sodnomdarjaa Ruuragc, Georgina Limon, Ginette Wilsden, Clare Browning, Donald P. King, Anna B. Ludi, Nicholas A. Lyons

**Affiliations:** a State Central Veterinary Laboratory, Ulaanbaatar, Mongolia; b General Authority for Veterinary Services, Government Building-IX, Enkhtaivan Avenue-16a, 3th Khoroo, Bayanzurkh District, Ulaanbaatar, Mongolia; c The Pirbright Institute, Ash Road, Pirbright, Woking, Surrey GU24 0NF, United Kingdom; d European Commission for the Control of Foot-and-Mouth Disease (EuFMD), Food and Agriculture of the United Nations, Rome, Italy

**Keywords:** Foot-and-mouth disease, Immunogenicity, Cattle, Sheep, Camels, Post-vaccination monitoring

## Abstract

Foot-and-mouth disease (FMD) is a high impact viral disease of livestock for which vaccines are extensively used in control. Mongolia has regular incursions of FMD virus that are typically limited to the eastern region although large epidemics are occasionally reported in the normally disease-free western areas. Vaccines are imported and form an important component of the control strategy. In 2015, post-vaccination monitoring guidelines were published by the FAO-OIE recommending approaches for assessing the appropriateness of imported vaccines including small-scale immunogenicity studies. This study used these recommended approaches to guide the use of vaccine adjuvant type and the need for a one or two dose primary course in the national control programme considering cattle, sheep and Bactrian camels and also whether these vaccines were appropriate for the FMD virus lineages considered high risk to Mongolia (A/ASIA/Sea-97; O/SEA/Mya-98; O/ME-SA/PanAsia; O/ME-SA/Ind-2001). The results of these immunogenicity studies indicated that in cattle and sheep, oil-adjuvanted vaccines led to higher and more persistent neutralisation titres that were satisfactory against the target lineages if a two-dose primary course was utilised. In contrast, aqueous-adjuvanted vaccines were associated with lower titres that likely required a booster after 3 months. Levels of antibodies in Bactrian camels were significantly lower although it is unknown how these may correlate with protection under experimental or field exposure conditions. The results of this study have implications for vaccine policy in Mongolia and suggest further studies on the role of Bactrian camels in the epidemiology of FMD are necessary to indicate if further research on FMD vaccines are needed in this species.

## Highlights

• FMD vaccines were independently assessed using published international guidelines.
• Highest titres were seen with oil-adjuvanted vaccines with a 2 dose primary course.
• Lower titres were seen with aqueous vaccines requiring a boost after 3 months.
• It is unknown how the lower titres observed in camels correlate with protection.
• The results have important implications for vaccine policy in Mongolia.

## 1 Introduction

Foot-and-mouth disease (FMD) is an endemic viral disease of cloven-hooved livestock that is present throughout large parts of the African and Asian continents. FMD vaccines are used extensively to control FMD with an estimated 2.38 billion doses used globally each year [1]. There are many FMD vaccine producers, mostly producing killed/inactivated vaccines that include either oil (usually Montanide**®** ISA25 or ISA206) or aqueous based (usually combined aluminium hydroxide [Al(OH)_3_ and saponin) adjuvants [2]. There are numerous factors that limit their effective use including low potency, poor antigenic match between the field and vaccine strains, reliance on a cold chain, short duration of action and population turnover limiting coverage [3]. Despite these limitations, vaccines have been used successfully for control and elimination of FMD virus in various settings [4], [5] and there is a huge global demand which is expected to increase with a growing global livestock population [1].

The Progressive Control Pathway for FMD (PCP-FMD) is a stepwise tool to assist endemic countries, developed and endorsed by the European Commission for the Control of Food-and-mouth disease (EuFMD), Food and Agriculture organisation of the United Nations (FAO) and World Organisation for Animal Health (OIE) [6]. Monitoring and evaluation of a disease control strategy is a key component of any control strategy and is fundamental to the PCP-FMD [7]. With the importance of vaccines in the control of FMD, in 2015 the FAO and OIE published Post Vaccination Monitoring (PVM) guidelines to advise countries on the principles and suggested procedures for monitoring various aspects of FMD vaccines [8]. This document includes assessments of quality, population-level immunity, coverage and effectiveness. Assessment of vaccine quality is done through small-scale immunogenicity studies.

Mongolia is a large, land-locked country in Asia bordered by Russia to the north and China to the south. According to the Mongolian Statistical Information Service, in 2018 there were approximately 4.4 million cattle, 30.6 million sheep, 27.1 million goats, and 0.46 million camels [9]. The majority of livestock are farmed extensively by nomadic herders making use of the extensive rangelands present in the country [10].

FMD is not believed to be endemic within Mongolia but repeated incursions occur in the Eastern region which occasionally spread into central and westerly areas which are typically FMD free where large epidemics can occur [11], [12]. Typically outbreaks are due to FMD viruses of either the O or A serotypes and disease incidence is usually highest in cattle followed by sheep and goats [11], [13]. Evidence of infection and/or disease has also been reported in wildlife and Bactrian camels [14], [15], [16]. A large upsurge of FMD cases was observed in 2017–18, with multiple lineages of serotype O being detected (O/SEA/Mya-98; O/ME-SA/PanAsia; O/ME-SA/Ind-2001) as well as a single lineage of serotype A (A/ASIA/Sea-97) [17]. During this episode, FMD virus was isolated for the first time from field cases among Bactrian camels [18]. Vaccination using imported vaccines was one of the major tactics to contain the epidemic. There was a need to conduct independent assessments of their appropriateness to inform future vaccination policy in Mongolia. The objectives of this study were to use the principles of the PVM guidelines to assess the immunogenicity of imported FMD vaccines in Mongolia during the 2017–2018 epidemic by comparing aqueous and oil adjuvanted vaccines in different species and to provide guidance on optimal schedules for use in a national programme of FMD vaccination.

## 2 Materials and methods

### 2.1 Study design

This study was conducted as part of the national FMD post-vaccination monitoring activities. Immunogenicity studies were based on those prescribed in section 3.3 of the FAO-OIE PVM Guidelines [8]. A series of six studies were conducted to assess the immunogenicity of aqueous and oil adjuvanted vaccines in cattle, sheep and Bactrian camels using one and two-dose primary courses (supplementary material A). In each study involving cattle or sheep, 12 animals were used that included two unvaccinated controls to act as sentinels for FMD virus exposure. Of the 10 vaccinated animals, 5 were given a second dose at 28 days post vaccination (dpv). Due to a lower availability of Bactrian camels, 6 were used for each study with 3 receiving a two-dose primary course (supplementary material A). Separate controls were used for each study. The vaccine manufacturer recommends a single dose primary course in immunologically naïve animals (i.e. with no previous exposure or vaccination or maternally derived antibodies) using either aqueous or oil adjuvanted vaccines so the timing of the second dose was consistent with recommendations in the literature [19]. Animals had serum samples taken at first vaccination (0dpv), 28dpv, 56dpv, 112dpv, and 180dpv. All animals were individually identified to ensure accurate follow-up and monitored for signs of clinical FMD.

### 2.2 Farm and animal selection

The study was undertaken in Orkhon Province (aimag) in the north-central part of the country. This province was selected as there has been no outbreak of FMD reported since 2001 and previous NSP antibody serosurveillance indicated no circulating virus. A single commercial farm was selected for all studies based on convenience and because they had all the species of interest for doing the study. All animals had serum samples taken prior to enrolment and were tested negative to non-structural protein antibodies. Animals were enrolled into the study if they were over two years old and were randomly assigned into groups (vaccine adjuvant type and 1 or 2 dose primary courses).

### 2.3 Vaccines

The multivalent vaccines used in this study were those used during the 2017–2018 epidemic. They were manufactured by ARRIAH (Vladimir, Russia) containing vaccine strains from the O/ME-SA/PanAsia and A/ASIA/Sea-97 lineages. These vaccines were purified to remove non-structural proteins. The aqueous vaccine was adjuvanted with aluminium hydroxide (Al(OH)_3_) and saponin (batch number 16) while the oil vaccine had Montanide® ISA 25 (batch number 21). The dose was given according to recommendations from the vaccine manufacturer (2 ml for cattle and camels, 1 ml for sheep) delivered in the middle part of the neck.

### 2.4 Sampling and serology

All serum samples were taken by venepucture using either the jugular or caudal (tail) vein depending on species and handling facilities present. Samples were transferred to the State Central Veterinary Laboratory (SCVL) in Ulan Bator on ice. Upon arrival in the laboratory, serum was separated and frozen at −20 °C until testing. All samples were tested for NSP antibodies using a commercially available ELISA kit (ID Screen® FMD NSP Competition, ID Vet).

Samples were subsequently shipped on ice to the World Reference Laboratory for FMD (WRLFMD) for testing by VNT as described previously [20]. Heterologous titres were derived using representative field isolates from Mongolia using the following lineages (WRLFMD code for isolate used in parentheses): A/ASIA/Sea-97 (A/MOG/1/2016); O/ME-SA/Ind-2001 (O/MOG/14/2017); O/SEA/Mya-98 (O/MOG/4/2015); and O/ME-SA/PanAsia (O/MOG/13/2017).

### 2.5 Data analysis

Non-parametric Wilcoxan rank sum and Fisher’s exact tests were used to compare the animal characteristics in the different groups. For analysis of VNT data, to account for right censoring of data and values falling between two dilutions, interval regression was used as described previously using the logarithm (base 10) of the reciprocal titres [21] but lower bounds for the lowest titrations were set to zero. A single multivariable model was run including lineage, days post vaccination, species (cattle, sheep or camels), number of doses (1 or 2), and vaccine adjuvant (aqueous or oil) as explanatory variables. To generate estimates at each time point for the different schedules and species, two interaction terms were included between these variables and the number of days post-vaccination. All analysis was done in Stata 15.0 (StataCorp LP, Texas, USA).

## 3 Results

The mean ages of animals recruited for the study were 2.8, 2.1, and 4.8 years for cattle, sheep and camels, respectively. The sex of the animals in each study was predominantly male except in camels (cattle 17/24, 70.8%; sheep 13/24, 54.2%; camels 5/12, 41.7%). The cattle were older in the oil (mean age = 3.2 years) versus aqueous (mean age = 2.3 years) adjuvanted vaccine groups (P = 0.019) with more males (12/12 [100%) cattle in the oil group, P = 0.005). There was no statistical evidence of a difference in these groups in sheep and camels.

There were no occurrences of suspected clinical FMD on the study farm and no animals seroconverted to NSP antibody during the study period (data not shown). Over the study period, a total of 5 cattle and 4 sheep exited the study due to other disease mortality between the 56 and 112 day sampling points (supplementary material B). Although in study number 1 (cattle receiving aqueous adjuvanted vaccine), both controls exited the study, the lack of seroconversion to NSP among animals receiving vaccine provide confidence that the observed changes in FMDV-specific antibody titres were induced by the vaccine, rather than exposure to FMD virus.

**Fig. 1:**
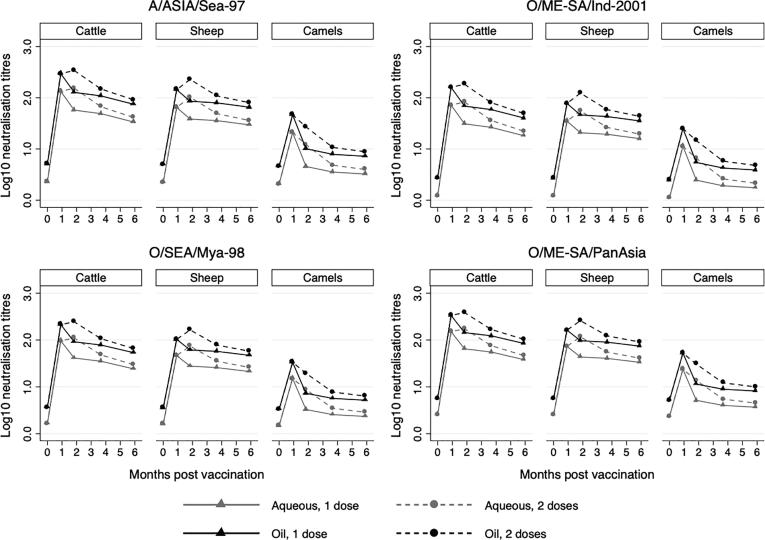
Post-vaccination neutralising titres against four field isolates of FMD virus isolated from Mongolia. Different species were vaccinated with either an aqueous or oil adjuvanted vaccine with some receiving a second dose at 28 days post vaccination. Data were generated through interval regression. For clarity, confidence intervals are not included but can be seen in supplementary material C.

Multivariable, interval regression models were used to analyse the neutralising antibody data (Table 1, Fig. 1). In cattle and sheep, the titres were above log_10_2.0 at 28dpv for all lineages when using an oil adjuvanted vaccine although a second dose was needed to maintain these levels at 56dpv (Fig. 1, supplementary material C). Analyses revealed evidence of higher titres against A/ASIA/Sea-97 compared to O/ME/Ind-2001 and O/SEA/Mya-98 although there was no evidence of a difference with O/ME-SA/PanAsia. After adjusting for lineage, adjuvant and dosing schedule, the neutralising titres measured in cattle were significantly higher than camels for all sampling time points. The titres in sheep were generally lower than cattle although there was no statistical evidence for a difference by 180dpv and based on non-overlapping 95% confidence intervals the titres in sheep were significantly higher than camels at all sampling points (supplementary material C). There was strong statistical evidence that the titres observed using the oil-based vaccine were higher than the aqueous vaccines at all time points and species. Although the two-dose primary course was also associated with higher titres, this effect did not persist and there was no evidence of a difference in titres by day 180 for any lineage.

**Table 1:** Average marginal effects estimated from multivariable interval regression comparing post vaccination neutralising antibody titres to different lineages of FMD virus isolated from field cases in Mongolia. Interaction terms were included to account for effect modification between species and dosing schedule (one or two dose) and days post vaccination. Unvaccinated controls not included in the model. All estimates given to two significant figures. Days post vaccination was included in the model as a categorical rather than a linear variable informed by likelihood ratio tests when (P < 0.001). CI = confidence interval.

Variable	Category	Marginal effect	95%CI	P-value
Lineage	A/ASIA/Sea-97	Baseline	–	–
	O/ME-SA/Ind-2001	−0.27	−0.33, −0.20	<0.001
	O/SEA/Mya-98	−0.14	−0.21, −0.079	<0.001
	O/ME-SA/PanAsia	0.054	−0.010, 0.12	0.10
Species	Cattle	Baseline	–	–
	Sheep	−0.14	−0.19, −0.086	<0.001
	Camels	−0.66	−0.73, −0.60	<0.001
Adjuvant	Aqueous	Baseline		
	Oil	0.35	0.30, 0.39	<0.001
Schedule	One dose	Baseline	–	–
	Two doses	0.087	0.042, 0.13	<0.001
Days post vaccination	0	Baseline	–	
	28	1.5	1.4, 1.5	<0.001
	56	1.3	1.3, 1.4	<0.001
	112	1.1	1.0, 1.2	<0.001
	180	0.99	0.92, 1.1	<0.001

**Fig. 2:**
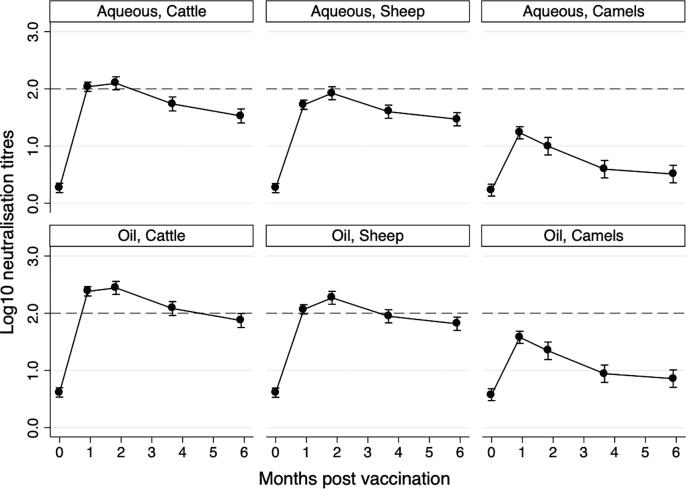
Post-vaccination neutralising titres for strains from relevant FMDV lineages in Mongolia (A/ASIA/Sea-97; O/ME-SA/Ind-2001; O/SEA/Mya-98; O/ME-SA/PanAsia) to inform the timing of a booster dose using aqueous and oil adjuvanted vaccines in cattle, sheep and camels. Only a two-dose primary course is presented. The dashed line at log_10_2.0 represents an estimated level that correlates with protection based on challenge studies in cattle [22].

Data were analysed to inform the timing of a booster dose using the different vaccine adjuvant types. Data for all lineages were analysed together and considered a two-dose primary course only. These data revealed that for the oil-based vaccine, titres were at or around log_10_2.0 until 6 months post initial vaccination in cattle but when using the aqueous based vaccine, titres dropped below log_10_2.0 by 3 months post vaccination (Fig. 2). In sheep, the observations with the oil-based vaccine were similar to cattle but with the aqueous adjuvanted vaccine the titres never reached log_10_2.0 and there was a significant drop between 2 and 3 months post vaccination. In camels the titres never reached these levels but had significantly dropped below the primary course level by 4 months post initial vaccination irrespective of vaccine adjuvant type.

## 4 Discussion

This study utilised recommended approaches to determine the immunogenicity of imported FMD vaccines in Mongolia and inform recommendations on their use in the field. Different vaccine adjuvants, species and one or two-dose primary courses were considered. Virus neutralisation tests (VNTs) were used to assess immunogenicity using Mongolian field strains from high-risk viral lineages (A/ASIA/Sea-97; O/ME-SA/Ind-2001; O/SEA/Mya-98; O/ME-SA/PanAsia). The results indicated that oil adjuvanted vaccines and a second dose at 28 days post vaccination (dpv) gave significantly higher titres adjusted for species and viral lineage. Titres in cattle were significantly higher than sheep, which in turn were significantly higher than camels. Neutralisation titres against the A/ASIA/Sea-97 and O/ME-SA/PanAsia lineages were similar although significantly higher than those against O/ME-SA/Ind-2001 and O/SEA/Mya-98. Results also indicated that a booster dose be administered at 6 months in cattle and sheep for oil-based vaccines and by 3 months for aqueous adjuvanted vaccines following a two-dose primary course.

Based on previous challenge studies in cattle using homologous strains of virus to the vaccine, and VNT performed at the same laboratory as the current study, titres above log_10_2.0 were associated with 95% clinical protection for strains of serotype A and O [22]. In cattle and sheep, oil adjuvanted vaccines induced neutralising titres above log_10_2.0 for all tested lineages although a second dose was required to maintain these above or around this level until 112–180 dpv. For aqueous adjuvanted vaccines, lower levels of antibody were induced which were consistently below the log_10_2.0 threshold between 112 and 180 dpv even if a second dose was included. These results indicate that to maintain these titres, a two-dose primary course must be included irrespective of the vaccine adjuvant consistent with previous field-based and experimental observations [23], [24].

The apparent superiority in titres observed using oil-adjuvanted vaccines in this study has been reported previously although one review suggested “little difference between the two adjuvants” although “there are many variations on the oil emulsion formulation” and cautioned against generalisations [24]. However, direct within-study comparison of adjuvant types on relative titres and the longevity of antibody response in the field are lacking in the scientific literature. Field and experimental studies from South America have suggested that oil-based vaccines can be given less frequently than aqueous with the former demonstrating satisfactory titres at 6 months post vaccination but the latter requiring boosters at 3 months [25], [26], [27]. However, one experimental study showed similar titres between Montanide ISA 206 and Al(OH)_3_-saponin adjuvanted vaccines up to 21 days post single vaccination in cattle [28]. Other immunogenicity studies comparing the same adjuvants up to 90 days post single vaccination in sheep, goats and cattle showed greater titres in those receiving the oil based vaccine [29], [30]. It has also been previously demonstrated in sheep that oil-adjuvanted vaccines induce titres for a longer time period compared to the aqueous equivalent [31], [32] consistent with the findings in this study, although another study showed no difference over two months post vaccination [33]. One study compared antibody kinetics for six months following a single dose of vaccine with either Montanide ISA 206, Montanide ISA 25 or Al(OH)_3_/saponin adjuvants and found only those vaccinated with the ISA 206 oil formulation maintained titres for the trial duration [34].

Differences in neutralising antibody responses post FMD vaccination in different species has been studied previously. These may be partly explained by the lower dose of vaccine that is typically used in small ruminants. A comparison of titres in cattle, sheep and goats following a single dose of a Montanide ISA 206 adjuvanted vaccines showed higher levels in sheep compared to cattle, with both being higher than goats [30]. This same study described sheep and goats as “late responders” to FMD vaccination with titres showing an “upward shift” between 30dpv and 60dpv. Another study observed a similar pattern using an oil-adjuvanted vaccine in sheep which observed antibody levels rising up to three months post vaccination although with an aqueous equivalent titres peaked at one month post vaccination [32]. This “late responder” pattern was not observed in the current study which demonstrated a peak in titres at 28dpv in those receiving a single dose and 56dpv in those receiving a second dose at 28dpv, irrespective of species or adjuvant type.

The persistently lower titres post vaccination in Bactrian camels compared to other species has not been demonstrated previously. One report indicated a relatively low response to first vaccination in dromedary camels followed by a stronger anamnestic response as measured by ELISA although low levels of neutralisation antibodies were seen even after two doses of vaccine [35]. The role of camels in the epidemiology of FMD is not clear although Bactrian camels have been shown to be relatively easier to infect compared to dromedary camels, and reports of clinical cases have been made previously in Mongolia with virus isolated from a field case in 2017 [16], [18], [36]. In Mongolia, the Bactrian species of camel predominates, and are included in FMD vaccination campaigns creating a need to know if vaccines are immunogenic and effective. Although the titres observed in this study were lower than the other species, it is unknown how these levels may correlate with protection from infection or disease. As a comparison, a challenge study of Bactrian camels and sheep reported lower virus neutralisation titres in the former species although this was only based on two animals per group and no statistical comparison was possible [36]. In a challenge study of dromedary camels, the reported neutralisation titre post inoculation was “modest” at between log_10_1.7 and log_10_1.8 [37]. The contrasting titres observed in camels may be partly or wholly explained by the different antibody structures within members of the *Camelidae* family that are known to lack a light chain although it is not clear how this may lead to relatively lower neutralising titres [38]. Alternatively, the antigen dose of vaccine may need to be modified in these species. However, their role in the epidemiology of FMD in Mongolia and other countries with significant camel populations should be clarified before investments are made in developing new or adapting existing vaccines.

There are numerous limitations to this study that need to be highlighted. Firstly, due to a lack of availability of the vaccine virus strains, neutralising titres were estimated using heterologous field strains. Therefore direct inferences on the quality of the imported batches of vaccine cannot be made, since both quality (i.e. antigen payload per dose) and match between the virus and field strains will affect heterologous titres. Moreover, the antigen payload, vaccine match (i.e. r_1_ values), concentration and specific types of adjuvant, presence of immuno-stimulant additives or antibiotics were also unknown. Secondly, the assumed level of neutralising antibodies that correlate with protection are based on experimental challenge studies giving high levels of virus at a single time point through artificial inoculation routes. Natural exposure is likely through multiple routes, at variable levels and for variable periods of time and so it is plausible that under field conditions different levels of antibody may correlate with protection. Thirdly, due to limitations in the study design it was not possible to evaluate the relative speeds of onset of immunity using the different adjuvants. This property is an important aspect to consider particularly when using reactive vaccination strategies although other factors such as the antigen payload are also important in this regard [39]. Furthermore, although suggestions on the timing of a booster dose were provided this was not objectively evaluated by testing antibody levels induced by booster vaccinations. Fourthly, this study only considered seronegative animals and it is well known that maternally derived antibodies interfere with the response to FMD vaccines [40], [41], [42], [43], [44]. Finally, as highlighted by Doel [42], there is a large variation in the types and quality of vaccine available globally so direct comparisons between reports in the literatures on FMD vaccines should be made with caution.

In conclusion, this study presented field-derived evidence of higher and more persistent antibody titres using an oil adjuvanted FMD vaccine compared to an aqueous equivalent and both required a two-dose primary course with boosters likely required at 6 or 3 months respectively to maintain titres at levels thought to be protective. The titres observed in Bactrian camels were significantly lower than cattle or sheep indicating further studies are needed establishing the role of this species in the epidemiology of FMD that will indicate a need for further research on FMD vaccines. These results have been used to inform FMD control policy in Mongolia and show the importance of post-vaccination monitoring and independent assessments of FMD vaccines to guide vaccination strategies and give confidence among stakeholders in the chosen approach.

## CRediT authorship contribution statement

**Gerelmaa Ulziibat:** Conceptualization, Investigation, Writing - original draft, Project administration, Visualization, Data curation. **Odonchimeg Maygmarsuren:** Investigation. **Bodisaikhan Khishgee:** Investigation. **Ganzorig Basan:** Supervision, Resources. **Batkhuyag Sandag:** Investigation. **Sodnomdarjaa Ruuragc:** Investigation. **Georgina Limon:** Writing - review & editing. **Ginette Wilsden:** Investigation. **Clare Browning:** Investigation. **Donald P. King:** Supervision, Resources, Funding acquisition. **Anna B. Ludi:** Conceptualization, Investigation, Resources, Supervision. **Nicholas A. Lyons:** Conceptualization, Methodology, Formal analysis, Writing - original draft, Visualization.

## Declaration of Competing Interest

The authors declare that they have no known competing financial interests or personal relationships that could have appeared to influence the work reported in this paper.
