# Supplementary material for: Immunogenicity of imported foot-and-mouth vaccines in different species in Mongolia
Source: Vaccine. 2020 Feb 11;38(7):1708–14. doi: 10.1016/j.vaccine.2019.12.053 (PMC7008245; doi:10.1016/j.vaccine.2019.12.053)
Supplement: Supplementary data 1 [file mmc1.docx]

Group composition for a series of studies to assess the immunogenicity of FMD vaccines in different species according to different adjuvant types and either a one or two dose primary course.

| **Study number** | **Species** | **Vaccine^a^** | **Second dose^b^** | **Number of subjects** |
| --- | --- | --- | --- | --- |
| 1 | Cattle | Aqueous | Yes | 5 |
|  |  |  | No | 5 |
|  |  | None | - | 2 |
| 2 | Cattle | Oil | Yes | 5 |
|  |  |  | No | 5 |
|  |  | None | - | 2 |
| 3 | Sheep | Aqueous | Yes | 5 |
|  |  |  | No | 5 |
|  |  | None | - | 2 |
| 4 | Sheep | Oil | Yes | 5 |
|  |  |  | No | 5 |
|  |  | None | - | 2 |
| 5 | Camels | Aqueous | Yes | 3 |
|  |  |  | No | 2 |
|  |  | None | - | 1 |
| 6 | Camels | Oil | Yes | 3 |
|  |  |  | No | 2 |
|  |  | None | - | 1 |

^a^ Vaccines used were manufactured by ARRIAH (Vladimir, Russia) containing vaccine strains from the O/ME-SA/PanAsia and A/ASIA/Sea-97 lineages. ^b^ Second dose given at 28 days post first vaccination
