# Supplementary material for: Immunogenicity of imported foot-and-mouth vaccines in different species in Mongolia
Source: Vaccine. 2020 Feb 11;38(7):1708–14. doi: 10.1016/j.vaccine.2019.12.053 (PMC7008245; doi:10.1016/j.vaccine.2019.12.053)
Supplement: Supplementary data 3 [file mmc3.docx]

Estimated neutralising antibody titres against different FMD viral strains following vaccination. The values are estimated based on a multivariable interval regression model including lineage, species, vaccine adjuvant type, inclusion of a second dose in the primary course and days post vaccination. dpv = days post vaccination, CI = confidence interval.

1. A/ASIA/Sea97

| **dpv** | **Vaccine type** | **Species** | **Dose** | **Titre** | **95%CI** | **P-value** |
| --- | --- | --- | --- | --- | --- | --- |
| 0 | Aqueous | Cattle | 1 | 0.36 | 0.27,0.45 | <0.001 |
| 28 | Aqueous | Cattle | 1 | 2.12 | 2.04,2.21 | <0.001 |
| 56 | Aqueous | Cattle | 1 | 1.76 | 1.64,1.88 | <0.001 |
| 112 | Aqueous | Cattle | 1 | 1.69 | 1.57,1.81 | <0.001 |
| 180 | Aqueous | Cattle | 1 | 1.53 | 1.41,1.65 | <0.001 |
| 0 | Aqueous | Cattle | 2 | 0.36 | 0.27,0.45 | <0.001 |
| 28 | Aqueous | Cattle | 2 | 2.12 | 2.04,2.21 | <0.001 |
| 56 | Aqueous | Cattle | 2 | 2.19 | 2.07,2.31 | <0.001 |
| 112 | Aqueous | Cattle | 2 | 1.82 | 1.70,1.95 | <0.001 |
| 180 | Aqueous | Cattle | 2 | 1.62 | 1.49,1.74 | <0.001 |
| 0 | Aqueous | Sheep | 1 | 0.35 | 0.26,0.44 | <0.001 |
| 28 | Aqueous | Sheep | 1 | 1.81 | 1.72,1.90 | <0.001 |
| 56 | Aqueous | Sheep | 1 | 1.59 | 1.47,1.71 | <0.001 |
| 112 | Aqueous | Sheep | 1 | 1.55 | 1.43,1.68 | <0.001 |
| 180 | Aqueous | Sheep | 1 | 1.47 | 1.34,1.60 | <0.001 |
| 0 | Aqueous | Sheep | 2 | 0.35 | 0.26,0.44 | <0.001 |
| 28 | Aqueous | Sheep | 2 | 1.81 | 1.72,1.90 | <0.001 |
| 56 | Aqueous | Sheep | 2 | 2.01 | 1.89,2.13 | <0.001 |
| 112 | Aqueous | Sheep | 2 | 1.69 | 1.57,1.81 | <0.001 |
| 180 | Aqueous | Sheep | 2 | 1.56 | 1.44,1.68 | <0.001 |
| 0 | Aqueous | Camels | 1 | 0.32 | 0.21,0.43 | <0.001 |
| 28 | Aqueous | Camels | 1 | 1.32 | 1.21,1.43 | <0.001 |
| 56 | Aqueous | Camels | 1 | 0.66 | 0.49,0.83 | <0.001 |
| 112 | Aqueous | Camels | 1 | 0.55 | 0.38,0.72 | <0.001 |
| 180 | Aqueous | Camels | 1 | 0.51 | 0.34,0.68 | <0.001 |
| 0 | Aqueous | Camels | 2 | 0.32 | 0.21,0.43 | <0.001 |
| 28 | Aqueous | Camels | 2 | 1.32 | 1.21,1.43 | <0.001 |
| 56 | Aqueous | Camels | 2 | 1.09 | 0.93,1.24 | <0.001 |
| 112 | Aqueous | Camels | 2 | 0.68 | 0.53,0.84 | <0.001 |
| 180 | Aqueous | Camels | 2 | 0.60 | 0.44,0.76 | <0.001 |
| 0 | Oil | Cattle | 1 | 0.70 | 0.61,0.79 | <0.001 |
| 28 | Oil | Cattle | 1 | 2.47 | 2.38,2.56 | <0.001 |
| 56 | Oil | Cattle | 1 | 2.11 | 1.99,2.22 | <0.001 |
| 112 | Oil | Cattle | 1 | 2.04 | 1.92,2.16 | <0.001 |
| 180 | Oil | Cattle | 1 | 1.87 | 1.75,2.00 | <0.001 |
| 0 | Oil | Cattle | 2 | 0.70 | 0.61,0.79 | <0.001 |
| 28 | Oil | Cattle | 2 | 2.47 | 2.38,2.56 | <0.001 |
| 56 | Oil | Cattle | 2 | 2.53 | 2.41,2.65 | <0.001 |
| 112 | Oil | Cattle | 2 | 2.17 | 2.04,2.30 | <0.001 |
| 180 | Oil | Cattle | 2 | 1.96 | 1.83,2.09 | <0.001 |
| 0 | Oil | Sheep | 1 | 0.70 | 0.61,0.79 | <0.001 |
| 28 | Oil | Sheep | 1 | 2.16 | 2.07,2.25 | <0.001 |
| 56 | Oil | Sheep | 1 | 1.93 | 1.81,2.05 | <0.001 |
| 112 | Oil | Sheep | 1 | 1.9 | 1.78,2.03 | <0.001 |
| 180 | Oil | Sheep | 1 | 1.82 | 1.69,1.95 | <0.001 |
| 0 | Oil | Sheep | 2 | 0.7 | 0.61,0.79 | <0.001 |
| 28 | Oil | Sheep | 2 | 2.16 | 2.07,2.25 | <0.001 |
| 56 | Oil | Sheep | 2 | 2.36 | 2.24,2.48 | <0.001 |
| 112 | Oil | Sheep | 2 | 2.04 | 1.91,2.16 | <0.001 |
| 180 | Oil | Sheep | 2 | 1.9 | 1.78,2.03 | <0.001 |
| 0 | Oil | Camels | 1 | 0.66 | 0.55,0.78 | <0.001 |
| 28 | Oil | Camels | 1 | 1.67 | 1.55,1.78 | <0.001 |
| 56 | Oil | Camels | 1 | 1.01 | 0.84,1.17 | <0.001 |
| 112 | Oil | Camels | 1 | 0.90 | 0.73,1.06 | <0.001 |
| 180 | Oil | Camels | 1 | 0.86 | 0.69,1.03 | <0.001 |
| 0 | Oil | Camels | 2 | 0.66 | 0.55,0.78 | <0.001 |
| 28 | Oil | Camels | 2 | 1.67 | 1.55,1.78 | <0.001 |
| 56 | Oil | Camels | 2 | 1.43 | 1.28,1.59 | <0.001 |
| 112 | Oil | Camels | 2 | 1.03 | 0.87,1.19 | <0.001 |
| 180 | Oil | Camels | 2 | 0.95 | 0.79,1.10 | <0.001 |

1. O/ME-SA/Ind-2001d

| **dpv** | **Vaccine type** | **Species** | **Dose** | **Titre** | **95%CI** | **P-value** |
| --- | --- | --- | --- | --- | --- | --- |
| 0 | Aqueous | Cattle | 1 | 0.09 | -0.00048,0.18 | 0.051 |
| 28 | Aqueous | Cattle | 1 | 1.86 | 1.77,1.95 | <0.001 |
| 56 | Aqueous | Cattle | 1 | 1.49 | 1.37,1.61 | <0.001 |
| 112 | Aqueous | Cattle | 1 | 1.42 | 1.30,1.54 | <0.001 |
| 180 | Aqueous | Cattle | 1 | 1.26 | 1.14,1.38 | <0.001 |
| 0 | Aqueous | Cattle | 2 | 0.09 | -0.00048,0.18 | 0.051 |
| 28 | Aqueous | Cattle | 2 | 1.86 | 1.77,1.95 | <0.001 |
| 56 | Aqueous | Cattle | 2 | 1.92 | 1.80,2.04 | <0.001 |
| 112 | Aqueous | Cattle | 2 | 1.56 | 1.43,1.68 | <0.001 |
| 180 | Aqueous | Cattle | 2 | 1.35 | 1.22,1.48 | <0.001 |
| 0 | Aqueous | Sheep | 1 | 0.086 | -0.0040,0.18 | 0.061 |
| 28 | Aqueous | Sheep | 1 | 1.54 | 1.45,1.63 | <0.001 |
| 56 | Aqueous | Sheep | 1 | 1.32 | 1.20,1.44 | <0.001 |
| 112 | Aqueous | Sheep | 1 | 1.29 | 1.16,1.41 | <0.001 |
| 180 | Aqueous | Sheep | 1 | 1.2 | 1.07,1.33 | <0.001 |
| 0 | Aqueous | Sheep | 2 | 0.086 | -0.0040,0.18 | 0.061 |
| 28 | Aqueous | Sheep | 2 | 1.54 | 1.45,1.63 | <0.001 |
| 56 | Aqueous | Sheep | 2 | 1.74 | 1.62,1.86 | <0.001 |
| 112 | Aqueous | Sheep | 2 | 1.42 | 1.30,1.54 | <0.001 |
| 180 | Aqueous | Sheep | 2 | 1.29 | 1.17,1.41 | <0.001 |
| 0 | Aqueous | Camels | 1 | 0.051 | -0.060,0.16 | 0.37 |
| 28 | Aqueous | Camels | 1 | 1.05 | 0.94,1.17 | <0.001 |
| 56 | Aqueous | Camels | 1 | 0.39 | 0.22,0.56 | <0.001 |
| 112 | Aqueous | Camels | 1 | 0.28 | 0.12,0.45 | 0.001 |
| 180 | Aqueous | Camels | 1 | 0.24 | 0.077,0.41 | 0.004 |
| 0 | Aqueous | Camels | 2 | 0.051 | -0.060,0.16 | 0.37 |
| 28 | Aqueous | Camels | 2 | 1.05 | 0.94,1.17 | <0.001 |
| 56 | Aqueous | Camels | 2 | 0.82 | 0.66,0.98 | <0.001 |
| 112 | Aqueous | Camels | 2 | 0.42 | 0.26,0.57 | <0.001 |
| 180 | Aqueous | Camels | 2 | 0.33 | 0.17,0.49 | <0.001 |
| 0 | Oil | Cattle | 1 | 0.44 | 0.35,0.53 | <0.001 |
| 28 | Oil | Cattle | 1 | 2.2 | 2.11,2.29 | <0.001 |
| 56 | Oil | Cattle | 1 | 1.84 | 1.72,1.96 | <0.001 |
| 112 | Oil | Cattle | 1 | 1.77 | 1.65,1.89 | <0.001 |
| 180 | Oil | Cattle | 1 | 1.61 | 1.48,1.73 | <0.001 |
| 0 | Oil | Cattle | 2 | 0.44 | 0.35,0.53 | <0.001 |
| 28 | Oil | Cattle | 2 | 2.2 | 2.11,2.29 | <0.001 |
| 56 | Oil | Cattle | 2 | 2.26 | 2.14,2.38 | <0.001 |
| 112 | Oil | Cattle | 2 | 1.9 | 1.78,2.03 | <0.001 |
| 180 | Oil | Cattle | 2 | 1.69 | 1.57,1.82 | <0.001 |
| 0 | Oil | Sheep | 1 | 0.43 | 0.34,0.52 | <0.001 |
| 28 | Oil | Sheep | 1 | 1.89 | 1.80,1.98 | <0.001 |
| 56 | Oil | Sheep | 1 | 1.66 | 1.54,1.78 | <0.001 |
| 112 | Oil | Sheep | 1 | 1.63 | 1.51,1.76 | <0.001 |
| 180 | Oil | Sheep | 1 | 1.55 | 1.42,1.68 | <0.001 |
| 0 | Oil | Sheep | 2 | 0.43 | 0.34,0.52 | <0.001 |
| 28 | Oil | Sheep | 2 | 1.89 | 1.80,1.98 | <0.001 |
| 56 | Oil | Sheep | 2 | 2.09 | 1.97,2.21 | <0.001 |
| 112 | Oil | Sheep | 2 | 1.77 | 1.65,1.89 | <0.001 |
| 180 | Oil | Sheep | 2 | 1.64 | 1.51,1.76 | <0.001 |
| 0 | Oil | Camels | 1 | 0.40 | 0.29,0.51 | <0.001 |
| 28 | Oil | Camels | 1 | 1.4 | 1.29,1.51 | <0.001 |
| 56 | Oil | Camels | 1 | 0.74 | 0.57,0.91 | <0.001 |
| 112 | Oil | Camels | 1 | 0.63 | 0.46,0.79 | <0.001 |
| 180 | Oil | Camels | 1 | 0.59 | 0.42,0.76 | <0.001 |
| 0 | Oil | Camels | 2 | 0.40 | 0.29,0.51 | <0.001 |
| 28 | Oil | Camels | 2 | 1.4 | 1.29,1.51 | <0.001 |
| 56 | Oil | Camels | 2 | 1.16 | 1.01,1.32 | <0.001 |
| 112 | Oil | Camels | 2 | 0.76 | 0.61,0.92 | <0.001 |
| 180 | Oil | Camels | 2 | 0.68 | 0.52,0.84 | <0.001 |

1. O/SEA/Mya-98

| **dpv** | **Vaccine type** | **Species** | **Dose** | **Titre** | **95%CI** | **P-value** |
| --- | --- | --- | --- | --- | --- | --- |
| 0 | Aqueous | Cattle | 1 | 0.22 | 0.13,0.31 | <0.001 |
| 28 | Aqueous | Cattle | 1 | 1.98 | 1.89,2.07 | <0.001 |
| 56 | Aqueous | Cattle | 1 | 1.62 | 1.50,1.74 | <0.001 |
| 112 | Aqueous | Cattle | 1 | 1.55 | 1.43,1.67 | <0.001 |
| 180 | Aqueous | Cattle | 1 | 1.38 | 1.26,1.51 | <0.001 |
| 0 | Aqueous | Cattle | 2 | 0.22 | 0.13,0.31 | <0.001 |
| 28 | Aqueous | Cattle | 2 | 1.98 | 1.89,2.07 | <0.001 |
| 56 | Aqueous | Cattle | 2 | 2.04 | 1.92,2.16 | <0.001 |
| 112 | Aqueous | Cattle | 2 | 1.68 | 1.55,1.81 | <0.001 |
| 180 | Aqueous | Cattle | 2 | 1.47 | 1.34,1.60 | <0.001 |
| 0 | Aqueous | Sheep | 1 | 0.21 | 0.12,0.30 | <0.001 |
| 28 | Aqueous | Sheep | 1 | 1.67 | 1.58,1.76 | <0.001 |
| 56 | Aqueous | Sheep | 1 | 1.44 | 1.32,1.56 | <0.001 |
| 112 | Aqueous | Sheep | 1 | 1.41 | 1.29,1.54 | <0.001 |
| 180 | Aqueous | Sheep | 1 | 1.33 | 1.20,1.46 | <0.001 |
| 0 | Aqueous | Sheep | 2 | 0.21 | 0.12,0.30 | <0.001 |
| 28 | Aqueous | Sheep | 2 | 1.67 | 1.58,1.76 | <0.001 |
| 56 | Aqueous | Sheep | 2 | 1.87 | 1.75,1.99 | <0.001 |
| 112 | Aqueous | Sheep | 2 | 1.55 | 1.42,1.67 | <0.001 |
| 180 | Aqueous | Sheep | 2 | 1.42 | 1.29,1.54 | <0.001 |
| 0 | Aqueous | Camels | 1 | 0.18 | 0.065,0.29 | 0.002 |
| 28 | Aqueous | Camels | 1 | 1.18 | 1.07,1.29 | <0.001 |
| 56 | Aqueous | Camels | 1 | 0.52 | 0.35,0.68 | <0.001 |
| 112 | Aqueous | Camels | 1 | 0.41 | 0.24,0.57 | <0.001 |
| 180 | Aqueous | Camels | 1 | 0.37 | 0.20,0.54 | <0.001 |
| 0 | Aqueous | Camels | 2 | 0.18 | 0.065,0.29 | 0.002 |
| 28 | Aqueous | Camels | 2 | 1.18 | 1.07,1.29 | <0.001 |
| 56 | Aqueous | Camels | 2 | 0.94 | 0.79,1.10 | <0.001 |
| 112 | Aqueous | Camels | 2 | 0.54 | 0.39,0.70 | <0.001 |
| 180 | Aqueous | Camels | 2 | 0.46 | 0.30,0.61 | <0.001 |
| 0 | Oil | Cattle | 1 | 0.56 | 0.47,0.65 | <0.001 |
| 28 | Oil | Cattle | 1 | 2.33 | 2.24,2.42 | <0.001 |
| 56 | Oil | Cattle | 1 | 1.96 | 1.84,2.08 | <0.001 |
| 112 | Oil | Cattle | 1 | 1.89 | 1.77,2.02 | <0.001 |
| 180 | Oil | Cattle | 1 | 1.73 | 1.61,1.85 | <0.001 |
| 0 | Oil | Cattle | 2 | 0.56 | 0.47,0.65 | <0.001 |
| 28 | Oil | Cattle | 2 | 2.33 | 2.24,2.42 | <0.001 |
| 56 | Oil | Cattle | 2 | 2.39 | 2.27,2.51 | <0.001 |
| 112 | Oil | Cattle | 2 | 2.03 | 1.90,2.16 | <0.001 |
| 180 | Oil | Cattle | 2 | 1.82 | 1.69,1.95 | <0.001 |
| 0 | Oil | Sheep | 1 | 0.56 | 0.47,0.65 | <0.001 |
| 28 | Oil | Sheep | 1 | 2.01 | 1.92,2.10 | <0.001 |
| 56 | Oil | Sheep | 1 | 1.79 | 1.67,1.91 | <0.001 |
| 112 | Oil | Sheep | 1 | 1.76 | 1.63,1.88 | <0.001 |
| 180 | Oil | Sheep | 1 | 1.67 | 1.54,1.80 | <0.001 |
| 0 | Oil | Sheep | 2 | 0.56 | 0.47,0.65 | <0.001 |
| 28 | Oil | Sheep | 2 | 2.01 | 1.92,2.10 | <0.001 |
| 56 | Oil | Sheep | 2 | 2.22 | 2.10,2.34 | <0.001 |
| 112 | Oil | Sheep | 2 | 1.89 | 1.77,2.01 | <0.001 |
| 180 | Oil | Sheep | 2 | 1.76 | 1.64,1.88 | <0.001 |
| 0 | Oil | Camels | 1 | 0.52 | 0.41,0.63 | <0.001 |
| 28 | Oil | Camels | 1 | 1.52 | 1.41,1.64 | <0.001 |
| 56 | Oil | Camels | 1 | 0.86 | 0.69,1.03 | <0.001 |
| 112 | Oil | Camels | 1 | 0.75 | 0.59,0.92 | <0.001 |
| 180 | Oil | Camels | 1 | 0.72 | 0.55,0.88 | <0.001 |
| 0 | Oil | Camels | 2 | 0.52 | 0.41,0.63 | <0.001 |
| 28 | Oil | Camels | 2 | 1.52 | 1.41,1.64 | <0.001 |
| 56 | Oil | Camels | 2 | 1.29 | 1.13,1.45 | <0.001 |
| 112 | Oil | Camels | 2 | 0.89 | 0.73,1.05 | <0.001 |
| 180 | Oil | Camels | 2 | 0.8 | 0.65,0.96 | <0.001 |

1. O/ME-SA/Panasia

| **dpv** | **Vaccine type** | **Species** | **Dose** | **Titre** | **95%CI** | **P-value** |
| --- | --- | --- | --- | --- | --- | --- |
| 0 | Aqueous | Cattle | 1 | 0.41 | 0.32,0.50 | <0.001 |
| 28 | Aqueous | Cattle | 1 | 2.18 | 2.09,2.27 | <0.001 |
| 56 | Aqueous | Cattle | 1 | 1.81 | 1.69,1.93 | <0.001 |
| 112 | Aqueous | Cattle | 1 | 1.74 | 1.62,1.87 | <0.001 |
| 180 | Aqueous | Cattle | 1 | 1.58 | 1.46,1.70 | <0.001 |
| 0 | Aqueous | Cattle | 2 | 0.41 | 0.32,0.50 | <0.001 |
| 28 | Aqueous | Cattle | 2 | 2.18 | 2.09,2.27 | <0.001 |
| 56 | Aqueous | Cattle | 2 | 2.24 | 2.12,2.36 | <0.001 |
| 112 | Aqueous | Cattle | 2 | 1.88 | 1.75,2.01 | <0.001 |
| 180 | Aqueous | Cattle | 2 | 1.67 | 1.54,1.80 | <0.001 |
| 0 | Aqueous | Sheep | 1 | 0.41 | 0.32,0.50 | <0.001 |
| 28 | Aqueous | Sheep | 1 | 1.86 | 1.77,1.95 | <0.001 |
| 56 | Aqueous | Sheep | 1 | 1.64 | 1.52,1.76 | <0.001 |
| 112 | Aqueous | Sheep | 1 | 1.61 | 1.48,1.73 | <0.001 |
| 180 | Aqueous | Sheep | 1 | 1.52 | 1.39,1.65 | <0.001 |
| 0 | Aqueous | Sheep | 2 | 0.41 | 0.32,0.50 | <0.001 |
| 28 | Aqueous | Sheep | 2 | 1.86 | 1.77,1.95 | <0.001 |
| 56 | Aqueous | Sheep | 2 | 2.07 | 1.95,2.19 | <0.001 |
| 112 | Aqueous | Sheep | 2 | 1.74 | 1.62,1.86 | <0.001 |
| 180 | Aqueous | Sheep | 2 | 1.61 | 1.49,1.73 | <0.001 |
| 0 | Aqueous | Camels | 1 | 0.37 | 0.26,0.48 | <0.001 |
| 28 | Aqueous | Camels | 1 | 1.37 | 1.26,1.49 | <0.001 |
| 56 | Aqueous | Camels | 1 | 0.71 | 0.54,0.88 | <0.001 |
| 112 | Aqueous | Camels | 1 | 0.60 | 0.44,0.77 | <0.001 |
| 180 | Aqueous | Camels | 1 | 0.57 | 0.40,0.73 | <0.001 |
| 0 | Aqueous | Camels | 2 | 0.37 | 0.26,0.48 | <0.001 |
| 28 | Aqueous | Camels | 2 | 1.37 | 1.26,1.49 | <0.001 |
| 56 | Aqueous | Camels | 2 | 1.14 | 0.98,1.30 | <0.001 |
| 112 | Aqueous | Camels | 2 | 0.74 | 0.58,0.90 | <0.001 |
| 180 | Aqueous | Camels | 2 | 0.65 | 0.50,0.81 | <0.001 |
| 0 | Oil | Cattle | 1 | 0.76 | 0.67,0.85 | <0.001 |
| 28 | Oil | Cattle | 1 | 2.52 | 2.43,2.62 | <0.001 |
| 56 | Oil | Cattle | 1 | 2.16 | 2.04,2.28 | <0.001 |
| 112 | Oil | Cattle | 1 | 2.09 | 1.97,2.21 | <0.001 |
| 180 | Oil | Cattle | 1 | 1.93 | 1.81,2.05 | <0.001 |
| 0 | Oil | Cattle | 2 | 0.76 | 0.67,0.85 | <0.001 |
| 28 | Oil | Cattle | 2 | 2.52 | 2.43,2.62 | <0.001 |
| 56 | Oil | Cattle | 2 | 2.59 | 2.47,2.71 | <0.001 |
| 112 | Oil | Cattle | 2 | 2.22 | 2.10,2.35 | <0.001 |
| 180 | Oil | Cattle | 2 | 2.02 | 1.89,2.14 | <0.001 |
| 0 | Oil | Sheep | 1 | 0.75 | 0.66,0.84 | <0.001 |
| 28 | Oil | Sheep | 1 | 2.21 | 2.12,2.30 | <0.001 |
| 56 | Oil | Sheep | 1 | 1.99 | 1.87,2.11 | <0.001 |
| 112 | Oil | Sheep | 1 | 1.95 | 1.83,2.08 | <0.001 |
| 180 | Oil | Sheep | 1 | 1.87 | 1.74,2.00 | <0.001 |
| 0 | Oil | Sheep | 2 | 0.75 | 0.66,0.84 | <0.001 |
| 28 | Oil | Sheep | 2 | 2.21 | 2.12,2.30 | <0.001 |
| 56 | Oil | Sheep | 2 | 2.41 | 2.29,2.53 | <0.001 |
| 112 | Oil | Sheep | 2 | 2.09 | 1.97,2.21 | <0.001 |
| 180 | Oil | Sheep | 2 | 1.96 | 1.84,2.08 | <0.001 |
| 0 | Oil | Camels | 1 | 0.72 | 0.61,0.83 | <0.001 |
| 28 | Oil | Camels | 1 | 1.72 | 1.61,1.83 | <0.001 |
| 56 | Oil | Camels | 1 | 1.06 | 0.89,1.23 | <0.001 |
| 112 | Oil | Camels | 1 | 0.95 | 0.78,1.12 | <0.001 |
| 180 | Oil | Camels | 1 | 0.91 | 0.74,1.08 | <0.001 |
| 0 | Oil | Camels | 2 | 0.72 | 0.61,0.83 | <0.001 |
| 28 | Oil | Camels | 2 | 1.72 | 1.61,1.83 | <0.001 |
| 56 | Oil | Camels | 2 | 1.49 | 1.33,1.64 | <0.001 |
| 112 | Oil | Camels | 2 | 1.08 | 0.93,1.24 | <0.001 |
| 180 | Oil | Camels | 2 | 1.0 | 0.84,1.16 | <0.001 |
